# Efficient Catalytic Removal of Polycyclic Aromatic Hydrocarbons from Produced Water Using Fe^2+^-Terpyridine-Modified Superparamagnetic Nanoparticles

**DOI:** 10.1021/acsomega.5c03394

**Published:** 2025-10-17

**Authors:** Saeed Bahadorikhalili, Hadi Nasrabadi

**Affiliations:** Texas A&M University, Department of Petroleum Engineering, 245 Spence St Building, 3116 TAMU, College Station, Texas 77843, United States

## Abstract

This study presents the development and application of a novel Fe^2+^-supported terpyridine-modified superparamagnetic iron oxide nanoparticle catalyst (Fe^2+^-Tpy@SPIONs) for the efficient removal of polycyclic aromatic hydrocarbons (PAHs) from produced water. The catalyst was synthesized through the coprecipitation of iron oxides, followed by surface functionalization with a terpyridine ligand to immobilize Fe^2+^, thereby enhancing its catalytic activity. Catalytic performance was optimized by adjusting pH, temperature, and catalyst dosage. Under the optimized conditions, the catalyst demonstrated superior efficiency in degrading PAHs, reducing total organic carbon (TOC) levels in real produced water samples, achieving an 82% reduction. Reusability tests indicated that the Fe^2+^-Tpy@SPIONs catalyst retained its catalytic activity and structural integrity over five consecutive cycles, with minimal performance loss, as confirmed by scanning electron microscopy (SEM) and Fourier transform infrared (FTIR) analyses. Control experiments further established that the catalytic activity required the presence of both Fe^2+^ and terpyridine on the SPIONs surface, as neither unmodified SPIONs nor Tpy@SPIONs alone resulted in significant PAH removal. This work underscores the potential of Fe^2+^-Tpy@SPIONs as an effective, reusable, and magnetically recoverable catalyst for large-scale environmental remediation, particularly for treating organic pollutants in wastewater produced by the oil and gas industry.

## Graphical abstract

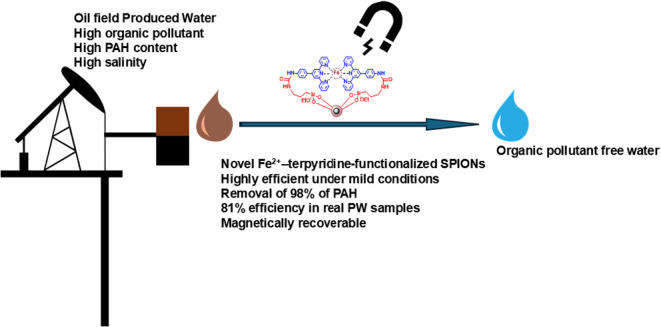

## 1 Introduction

Polycyclic aromatic hydrocarbons (PAHs) are a class of organic compounds characterized by multiple fused aromatic rings, which impart significant chemical stability and resistance to degradation.^1^ These compounds are primarily generated during the incomplete combustion of organic materials, such as coal, oil, and gas. Due to their hydrophobic nature, PAHs tend to accumulate in sediments and aquatic environments, leading to long-term environmental contamination and associated health risks.^2^

In the oil and gas industry, PAHs are a significant concern, particularly in produced water (PW), which refers to the wastewater generated during oil and gas extraction.^3–6^ In fact, PW is one of the largest waste streams produced by the oil and gas industry, constituting up to 98% of the total fluids extracted from mature oil wells.^7^ This byproduct consists of a complex mixture of dissolved hydrocarbons, salts, heavy metals, and polycyclic aromatic hydrocarbons. The global volume of produced water is staggering, with estimates ranging from 20 to 30 billion barrels annually in the United States and over 240 billion barrels worldwide.^8–10^ The annual volume of PW is substantially greater than that of other types of liquid hazardous waste.^11^ One of the major environmental concerns associated with PAHs in PW is their persistence and toxicity. Additionally, PW poses significant environmental and regulatory challenges, primarily due to its extremely high salinity, which can be up to ten times greater than that of seawater. PAHs are recognized as carcinogenic, mutagenic, and teratogenic, meaning they can cause cancer, genetic mutations, and birth defects in living organisms.^5,12,13^ Their long-term presence in aquatic environments poses risks not only to aquatic life but also to humans who consume contaminated water or seafood.^14^ Furthermore, the stability of PAHs in PW enables their spread over large areas, contaminating soils, groundwater, and surface water bodies.^15–18^ This makes their removal essential for environmental protection, particularly in regions where oil and gas extraction is a significant industrial activity.

The high salinity of produced water, combined with its complex mixture of hydrocarbons, salts, heavy metals, and PAHs, poses significant challenges for treatment. Salinity interferes with conventional methods, reducing their effectiveness and increasing costs. Effective treatment is critical to minimizing environmental impact and enabling the reuse of water in industrial or agricultural applications. Traditional methods, such as physical separation and chemical treatments, often fail to fully eliminate PAHs, underscoring the need for advanced technologies to address these challenges.^19–21^ Advanced methods, such as catalytic degradation, are emerging as more effective solutions. A number of methods including developing photobioreactors,^22^ indigenous oil degrading microbial communities,^23^ and developed filtration methods^24^ have been suggested for the removal of these contaminants from PW. The development and application of these technologies are crucial for mitigating the environmental impacts of oil and gas operations and for ensuring sustainable water management practices within industry.

Superparamagnetic iron oxide nanoparticles (SPIONs) are a class of nanomaterials that exhibit unique magnetic properties, making them highly versatile for various applications, particularly in catalysis and environmental remediation. These nanoparticles, typically composed of iron oxide (Fe_3_O_4_ or γ-Fe_2_O_3_), are characterized by their superparamagnetism at specific sizes, usually below 20 nm. Superparamagnetism refers to the ability of SPIONs to exhibit strong magnetization in the presence of an external magnetic field but lose this magnetization once the field is removed. This reversible magnetic behavior distinguishes SPIONs from ferromagnetic materials, which retain their magnetization.^25,26^ Due to this property, SPIONs can be easily manipulated using a magnetic field, enabling efficient recovery and reuse, which is particularly advantageous in catalytic processes and environmental remediation efforts. SPIONs have attracted significant attention in catalysis and environmental applications due to their distinctive combination of properties. One of their primary advantages in environmental remediation is their ease of separation. After a catalytic reaction, the nanoparticles can be magnetically recovered from the reaction mixture, eliminating the need for filtration or centrifugation.^27–30^ This simplification not only streamlines the process but also enhances cost-effectiveness and sustainability by enabling repeated use of the same catalytic material. Additionally, SPIONs offer significant potential for surface modification.^31,32^ Their surfaces can be functionalized with various organic or inorganic molecules, enabling fine-tuning of their catalytic properties. This flexibility allows SPIONs to be tailored for specific reactions, enhancing both their catalytic efficiency and selectivity. In catalysis, SPIONs are particularly effective as catalyst supports due to their large surface area, ease of surface modification, and stability. When functionalized with other catalytic species, such as noble metals or ligands, SPIONs become even more versatile, enabling them to catalyze a broader range of chemical transformations.^33,34^ The ability to modify SPIONs with ligands or other catalytic groups enhances their reactivity and selectivity for specific pollutants or reaction pathways.

In advanced applications, surface modification with ligands, such as terpyridine, can generate SPION-based catalytic systems that coordinate with transition metal ions like Fe^2+^, further enhancing their catalytic performance.^35,36^ These functionalized SPIONs not only improve the efficiency of pollutant degradation but also enhance the stability of the nanoparticles under harsh reaction conditions, making them durable catalysts for repeated use. The combination of magnetic recoverability, surface modifiability, and catalytic activity makes SPIONs a highly attractive option for both industrial catalysis and environmental remediation, where the demand for efficient, reusable, and adaptable catalysts is rapidly increasing.

The Fenton reaction is a powerful oxidation process that involves the generation of hydroxyl radicals (^•^OH) through the reaction of hydrogen peroxide (H_2_O_2_) with ferrous ions (Fe^2+^).^37–41^ This process is highly effective due to the strong oxidative potential of hydroxyl radicals, which are among the most reactive species.^42,43^ The mechanism starts with the reaction of Fe^2+^ with H_2_O_2_, producing Fe^3+^ and a hydroxyl radical, which then reacts with organic contaminants, breaking down complex molecules into smaller, less harmful compounds. Fe^3+^ can subsequently be reduced back to Fe^2+^ by organic compounds or other reducing agents, completing the catalytic cycle and enabling the process to continue.^44–46^ The oxidative power of the Fenton reaction stems from the unmatched reactivity of hydroxyl radicals, which can nonselectively attack a wide range of organic molecules, leading to their fragmentation and eventual mineralization into carbon dioxide and water. This chain reaction makes the Fenton process particularly effective for degrading persistent organic pollutants such as PAHs, dyes, and other hazardous organic substances commonly found in industrial wastewater. In environmental remediation, the Fenton process has been widely applied to treat contaminated water and soil, especially for the removal of industrial pollutants like PAHs, which are known for their stability and toxicity.^47–50^ Fenton-based oxidation effectively breaks down these organic pollutants, reducing their environmental impact and facilitating the safer disposal of industrial effluents. The high efficiency and relatively low cost of the Fenton reaction, coupled with its ability to degrade a broad spectrum of organic contaminants, make it an essential tool in modern environmental engineering for organic pollution control.^51^

Despite significant advancements in environmental catalysis, a notable gap remains in the literature concerning the development of convenient methods for PAH removal from produced water (PW). Although SPIONs have been extensively explored for various environmental applications, including pollutant degradation, few studies have examined their catalytic potential and efficiency in complex wastewater systems such as PW. Existing research predominantly focuses on traditional Fenton-like catalysts or alternative nanoparticle systems, without fully exploring the synergistic potential of integrating iron­(II) ions with terpyridine-modified SPIONs. This gap leaves an incomplete understanding of how this unique combination could not only enhance the catalytic breakdown of persistent organic pollutants like PAHs but also improve the stability and reusability of catalysts in harsh industrial wastewater conditions. Addressing this gap is critical, as produced water is a major source of environmental contamination, and the development of highly effective, recyclable catalysts is essential for sustainable remediation practices.

This work presents a novel and significant advancement in the treatment of produced water through the development of Fe^2+^-supported terpyridine-modified superparamagnetic iron oxide nanoparticles (SPIONs) as a highly efficient catalyst for the removal of polycyclic aromatic hydrocarbons (PAHs). The key innovation lies in the use of terpyridine ligands to immobilize Fe^2+^ ions, a unique approach that has not previously been applied in produced water treatment. This linkage enhances catalytic activity and provides an intriguing solution to the persistent challenge of PAH degradation. The study evaluates the effectiveness of this novel catalyst through detailed kinetic studies and mechanistic analyses under simulated produced water conditions. Key aspects such as PAH removal efficiency, catalyst reusability, and stability across multiple cycles are examined, along with the impact of variables such as temperature, pH, and pollutant concentration. Advanced characterization methods reveal critical structural insights both before and after catalysis. This innovative approach not only improves the efficiency of PAH removal but also reduces treatment costs, offering substantial environmental and economic benefits. The novel Fe^2+^-terpyridine linkage establishes a new frontier in nanoparticle-based remediation technologies, with broad implications for addressing organic contaminants across various industrial sectors. Unlike conventional Fenton-like catalysts that often require strongly acidic conditions and exhibit poor recyclability, Fe^2+^-Tpy@SPIONs operate efficiently under mild conditions, maintain structural integrity over multiple cycles, and can be magnetically recovered with minimal loss of activity. These unique features distinguish our system as a practical and scalable solution for the catalytic removal of persistent organic contaminants from produced water.

## 2 Experimental Methods

### 2.1 Materials and Reagents

Iron­(II) chloride (FeCl_2_·4H_2_O) and iron­(III) chloride (FeCl_3_·6H_2_O) were obtained from Sigma-Aldrich (purity ≥ 98%) and used as received for the synthesis of superparamagnetic iron oxide nanoparticles (SPIONs). Tetraethyl orthosilicate (TEOS, 98% purity, Sigma-Aldrich) served as the silica source for encapsulating the nanoparticles. Triethoxysilyl propyl isocyanate (97% purity, Sigma-Aldrich) was used for the surface modification of the SiO_2_-coated SPIONs. The terpyridine ligand, 4-([2,2′:6′,2’’-terpyridin]-4′-yl)­aniline (97% purity, Sigma-Aldrich), was employed to functionalize the nanoparticles. FeCl_2_ (98% purity, Sigma-Aldrich) was used for the immobilization of Fe^2+^ ions on the terpyridine-modified SPIONs. Hydrogen peroxide (30%, Fisher Scientific) was utilized in catalytic activity experiments. All solvents, including ethanol, methanol, and deionized water, were of analytical grade and used without further purification. Nitrogen gas (99.99% purity) was used during the synthesis process to maintain an inert atmosphere, as required. All reagents were handled following standard laboratory protocols without additional purification steps, unless otherwise specified.

### 2.2 Fe^2+^-Tpy@SPIONs Catalyst Synthesis

#### 2.2.1 SPION Synthesis

The superparamagnetic iron oxide nanoparticles (SPIONs) were synthesized using a coprecipitation method.^52^ FeCl_2_·4H_2_O (2 mmol) and FeCl_3_·6H_2_O (4 mmol) were dissolved in 100 mL of deionized water under a nitrogen atmosphere to prevent oxidation. The solution was heated to 80 °C with vigorous stirring, and aqueous ammonia solution was then slowly added dropwise to adjust the pH to approximately 10. This induced the coprecipitation of Fe^3+^ and Fe^2+^ ions as iron oxide (Fe_3_O_4_) nanoparticles. The mixture was stirred for an additional hour at 80 °C to ensure complete nanoparticle formation. After the reaction, the black precipitate (SPIONs) was magnetically separated, washed multiple times with deionized water and ethanol to remove any unreacted reagents, and then dried under vacuum at room temperature.

#### 2.2.2 Encapsulation with SiO_2_

To improve stability and provide a surface for further modification, the SPIONs were encapsulated with a silica shell using the Stöber method. The SPIONs (500 mg) were dispersed in a mixture of 80 mL ethanol, 20 mL deionized water, and 2 mL ammonia solution via ultrasonication for 30 min to ensure a homogeneous suspension. Tetraethyl orthosilicate (TEOS, 0.5 mL) was then added dropwise to the solution under continuous stirring. The reaction proceeded for 6 h at room temperature to form a uniform silica layer around the SPIONs. After the reaction, the SiO_2_-coated SPIONs were collected by magnetic separation, washed with ethanol and deionized water, and dried under vacuum.

#### 2.2.3 Surface Modification

The surface of the SiO_2_-encapsulated SPIONs was then modified with triethoxysilyl propyl isocyanate to introduce isocyanate functional groups for subsequent ligand attachment. In a typical reaction, 500 mg of SiO_2_-coated SPIONs were dispersed in 50 mL of dry toluene via ultrasonication. Triethoxysilyl propyl isocyanate (0.3 mL) was added to the mixture, and the reaction was refluxed at 80 °C for 12 h under a nitrogen atmosphere. After the reaction, the functionalized SPIONs were magnetically separated, washed with toluene and ethanol, and dried under vacuum. The isocyanate-functionalized surface was now prepared for the attachment of terpyridine ligands.

#### 2.2.4 Terpyridine Functionalization

To introduce the terpyridine ligand onto the SPIONs, 4-([2,2′:6′,2’’-terpyridin]-4′-yl)­aniline (200 mg) was dissolved in 50 mL of DMF and added to the isocyanate-modified SPIONs, which had been dispersed in DMF and sonicated for 30 min. The reaction mixture was stirred at room temperature for 24 h, allowing the terpyridine ligands to covalently bond to the SPION surface through the reaction between the isocyanate groups and the amine group on the terpyridine. After the reaction, the terpyridine-modified SPIONs (Tpy@SPIONs) were magnetically separated, thoroughly washed with ethanol and deionized water to remove any unbound ligands, and dried under vacuum.

#### 2.2.5 Fe^2+^ Immobilization

The final step in the catalyst synthesis involved the immobilization of Fe^2+^ ions onto the terpyridine-functionalized SPIONs. For this, the Tpy@SPIONs (500 mg) were dispersed in 50 mL of ethanol, and a solution of FeCl_2_ (0.1 mmol in 10 mL ethanol) was added dropwise to the dispersion under stirring. The reaction was maintained at room temperature for 12 h, allowing the Fe^2+^ ions to coordinate with the terpyridine ligands on the nanoparticle surface. After the reaction, the Fe^2+^-supported terpyridine-modified SPIONs (Fe^2+^-Tpy@SPIONs) were magnetically separated, washed with ethanol and deionized water, and dried under vacuum. The resulting catalyst, Fe^2+^-Tpy@SPIONs, was then ready for testing in the catalytic degradation of PAHs in produced water.

### 2.3 Reaction Setup

The catalytic activity of Fe^2+^-Tpy@SPIONs was tested in a batch reactor system to evaluate the degradation of PAHs in simulated produced water. The reactor consisted of a 250 mL glass round-bottom flask equipped with a magnetic stirrer to ensure uniform mixing of the reactants. Simulated produced water was prepared by dissolving fluorene, as a model PAH, in deionized water at a concentration of 50 ppm. The pH of the solution was adjusted to the desired value using diluted NaOH or HCl solutions.

### 2.4 Reaction Conditions

The catalytic degradation reactions were carried out under various conditions to evaluate the performance of Fe^2+^-Tpy@SPIONs. For each catalytic test, a fixed amount of Fe^2+^-Tpy@SPIONs was introduced into the batch reactor. The catalyst concentration was maintained at 0.5 g/L to ensure sufficient catalytic activity while allowing for efficient magnetic recovery of the nanoparticles after the reaction. The reaction temperature was kept at 25 °C (room temperature) unless otherwise specified, with the system constantly stirred at 300 rpm to maintain proper suspension of the nanoparticles. The oxidation reaction was initiated by adding 30% hydrogen peroxide (H_2_O_2_) as the oxidizing agent at a molar ratio of H_2_O_2_ to PAHs of 5:1. The reaction proceeded for 2 h, during which samples were withdrawn at regular intervals (15, 30, 60, and 120 min) to monitor the degradation of PAHs.

### 2.5 Control Experiments

To establish the effectiveness of Fe^2+^-Tpy@SPIONs, control experiments were conducted in parallel, including the following:

1. **Blank Experiment (No Catalyst):** A reaction was performed under identical conditions without the addition of any catalyst to assess the natural degradation of PAHs in the presence of hydrogen peroxide alone.
2. **Unmodified SPIONs:** A second control experiment was conducted using unmodified SPIONs (without terpyridine or Fe^2+^ immobilization) to evaluate the contribution of surface modification and the role of Fe^2+^ in the catalytic activity.
3. **Terpyridine-Modified SPIONs Without Fe** ^**2+**^ **:** This control assessed the catalytic performance of the terpyridine-modified SPIONs in the absence of Fe^2+^ ions to determine the specific role of Fe^2+^ in the degradation process.

The results of the control experiments were compared with those of Fe^2+^-Tpy@SPIONs to demonstrate the superior catalytic efficiency of the modified nanoparticles in degrading PAHs.

### 2.6 Analysis of Real Produced Water (PW) Samples

To evaluate the effectiveness of Fe^2+^-Tpy@SPIONs under real-world conditions, the catalytic degradation of organic contaminants in actual produced water (PW) samples was tested. For clarification, it should be mentioned that the term real PW refers specifically to the produced water samples collected from an actual oil and gas extraction site, prior to any treatment. On the other hand, treated PW refers to the same real produced water samples after undergoing catalytic treatment using the Fe^2+^-Tpy@SPIONs catalyst under optimized conditions. The total organic carbon (TOC) content was measured using a Sievers InnovOx Laboratory TOC Analyzer before and after treatment with the catalyst to quantify the reduction of organic matter, including PAHs and other organic pollutants. Real PW samples were obtained from an oil and gas extraction site. The initial TOC content of the produced water was determined using a TOC analyzer, which quantifies the organic carbon present by oxidizing the sample and measuring the resulting carbon dioxide. The amount of hydrogen peroxide added was based on a 5:1 molar ratio relative to the total organic carbon, assuming that each mole of TOC corresponds to one mole of carbon. TOC was measured at 637 mg/L, which corresponds to approximately 0.053 mol C/L. pH of the sample was measured as 6.00 and used in the reaction without adjusting the pH. The specifications of the PW samples are presented in Table 1.

**Table 1:** Specification of real PW sample

quantity	pH	density^a^	TSS^b^	TDS^c^	TOC
measured	6.00	1.2371 g/mL	510.0 mg/L	300,700 mg/L	637 mg/L

a Density at 20 °C.

b Total suspended solids.

c Total dissolved solids.

### 2.7 Catalytic Treatment of PW

The filtered PW samples (100 mL) were placed in a batch reactor, and the Fe^2+^-Tpy@SPIONs catalyst was added at a concentration of 0.5 g/L. The oxidation reaction was initiated by adding 30% hydrogen peroxide at a molar ratio of 5:1 relative to the number of moles of carbon. The mixture was stirred at room temperature (25 °C) for 2 h to facilitate the degradation of organic contaminants. Throughout the reaction, samples were taken at regular intervals to monitor the progress of TOC reduction. The sample, after reaction in the presence of hydrogen peroxide and the catalyst was considered as the treated sample.

### 2.8 TOC Measurement

Before and after the catalytic reaction, the TOC of the PW samples were measured using a TOC analyzer. This method involves oxidizing the organic carbon in the sample to carbon dioxide, which is then quantified by an infrared detector. The difference in TOC levels before and after treatment indicates the extent of organic matter degradation. The percentage reduction in TOC was calculated to assess the efficiency of the catalyst in breaking down organic pollutants in the real PW samples.

### 2.9 Results Interpretation

The TOC reduction after exposure to the Fe^2+^-Tpy@SPIONs catalyst provided a direct measure of the catalyst’s effectiveness in degrading the complex mixture of organic contaminants present in real produced water. A significant decrease in TOC indicates successful degradation of PAHs and other organic compounds.

### 2.10 Reusability and Stability Tests

The reusability and stability of the Fe^2+^-Tpy@SPIONs catalyst were evaluated over multiple catalytic cycles to assess its potential for long-term application in PAH degradation. After each catalytic reaction, the catalyst was recovered using magnetic separation, a key advantage of superparamagnetic nanoparticles. A strong external magnet was placed near the reactor to attract the Fe^2+^-Tpy@SPIONs to the side, allowing the clear supernatant to be decanted carefully. The recovered nanoparticles were then washed thoroughly with deionized water and ethanol to remove any residual reactants or byproducts. The cleaned catalyst was dried under vacuum at room temperature before reuse in subsequent catalytic tests.

For each cycle, a fresh batch of PAH-contaminated simulated produced water was prepared, and the same procedure for the catalytic activity test was followed as described earlier. The concentration of Fe^2+^-Tpy@SPIONs remained consistent across cycles (0.5 g/L), and the same reaction conditions, including temperature, pH, PAH concentration, and hydrogen peroxide dosage, were maintained to ensure consistency in the evaluation.

To evaluate the stability of the catalyst, catalytic activity was monitored across five cycles. The degradation efficiency of PAHs was measured after each cycle, and any decrease in performance was noted. The structural and chemical stability of the Fe^2+^-Tpy@SPIONs after multiple uses was assessed using various characterization techniques. For instance, scanning electron microscopy (SEM) was used to examine changes in nanoparticle morphology, while Fourier transform infrared spectroscopy (FTIR) was employed to detect any changes in surface composition and ligand coordination.

By comparing the catalytic activity of Fe^2+^-Tpy@SPIONs across multiple cycles, the reusability and long-term stability of the catalyst were confirmed. The ability to maintain high PAH degradation efficiency over repeated cycles, with minimal loss of catalytic performance or structural integrity, demonstrated the robustness of the Fe^2+^-Tpy@SPIONs as a sustainable solution for environmental remediation.

## 3 Results and Discussion

This paper presents a comprehensive investigation into the development and application of Fe^2+^-supported terpyridine-modified SPIONs as a catalytic system for the efficient removal of PAHs from produced water. The synthesis of the catalyst begins with the preparation of SPIONs through the coprecipitation of iron salts, specifically FeCl_2_ and FeCl_3_, in a basic medium. This method ensures the formation of well-defined iron oxide nanoparticles with superparamagnetic properties. Following this, the SPIONs are encapsulated in a silica (SiO_2_) shell using tetraethyl orthosilicate (TEOS) as a precursor, which enhances their stability and provides a suitable surface for further modification.

Once encapsulated, the surface of the SiO_2_-coated SPIONs is further functionalized using triethoxysilyl propyl isocyanate, which introduces reactive isocyanate groups that facilitate subsequent ligand attachment. The next step involves modifying the surface with 4-([2,2′:6′,2’’-terpyridin]-4′-yl)­aniline, a terpyridine-based ligand known for its ability to coordinate with metal ions and enhance catalytic properties. This ligand plays a crucial role in immobilizing Fe^2+^ ions, which are introduced in the final step by reacting the terpyridine-modified nanoparticles with FeCl_2_. The Fe^2+^ ions are then supported on the surface of the nanoparticles, creating a highly active catalytic system capable of efficiently degrading PAHs through oxidative pathways. The resulting Fe^2+^-supported terpyridine-modified SPIONs catalyst (Fe^2+^-Tpy@SPIONs) combines the advantages of magnetic recoverability, enhanced catalytic activity, and surface stability, making them a promising catalyst for environmental applications, particularly in the treatment of produced water contaminated with persistent organic pollutants such as PAHs. The synthesis steps of the catalyst are presented in Scheme 1.

**Scheme 1:**
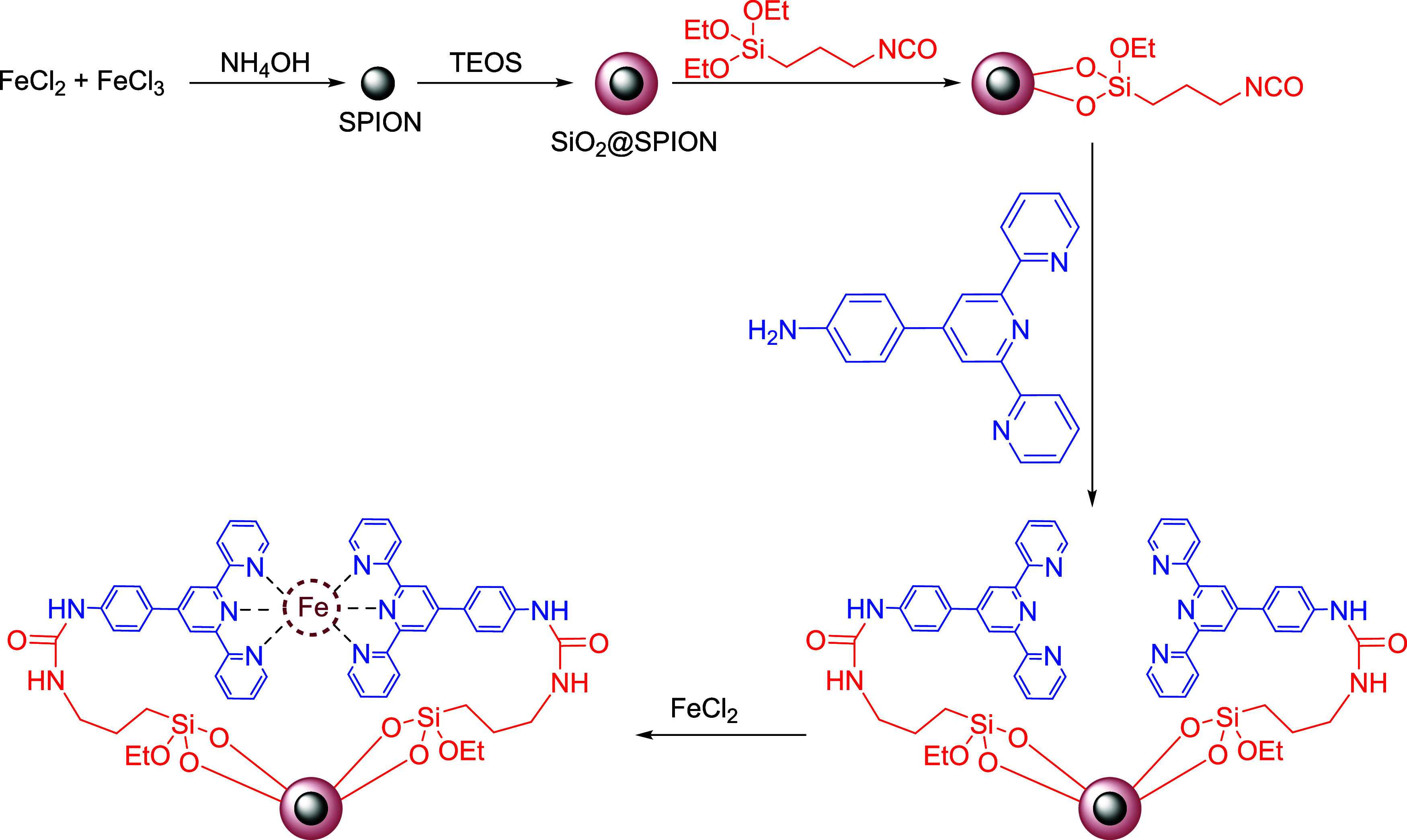
Synthesis Steps of Fe^2+^-Tpy@SPIONs Catalyst

The SEM analysis of the Fe^2+^-Tpy@SPIONs catalyst revealed that the nanoparticles exhibit a uniform spherical morphology with a well-defined structure. The average particle size was determined to be approximately 25 nm, consistent across various samples, indicating a successful synthesis and encapsulation process. This spherical shape and small particle size are critical for providing a large surface area and enhancing the catalytic performance of the nanoparticles. Additionally, the size distribution observed in SEM images was confirmed through dynamic light scattering (DLS) measurements, further validating the average size of 25 nm. The close agreement between SEM and DLS results suggests that the particles remain well-dispersed and retain their nanoscale dimensions, which is essential for ensuring efficient catalytic activity and stability during the degradation of PAHs in produced water. DLS and SEM results are presented in Figure 1a,1b, respectively.

**Figure 1:**
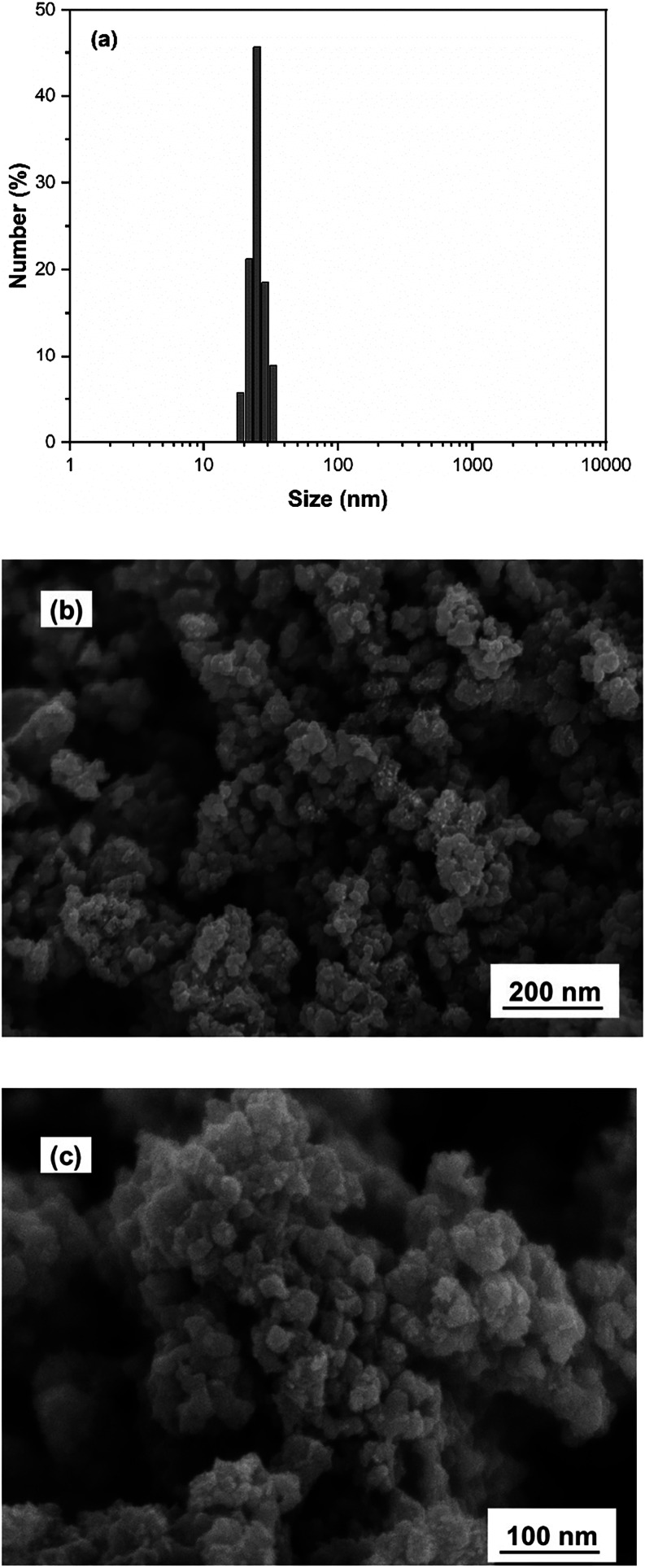
(a) DLS curve and (b, c) SEM image of Fe^2+^-Tpy@SPIONs catalyst with two different magnifications.

X-ray diffraction (XRD) analysis was performed to determine the crystalline structure of the Fe^2+^-Tpy@SPIONs catalyst, with results shown in Figure 2a. The diffraction pattern exhibits sharp and well-defined peaks at 2θ values of 18.3, 30.1, 35.5, 43.1, 53.4, 57.0, and 62.6°, which correspond to the (111), (220), (311), (400), (422), (511), and (440) crystal planes, respectively. These reflections are in excellent agreement with the standard pattern of magnetite (Fe_3_O_4_), confirming the successful formation of a highly crystalline spinel structure. The dominance of the (311) peak at 35.5° further highlights the phase purity and crystallinity of the synthesized nanoparticles. The absence of impurity peaks suggests that the functionalization steps did not disrupt the core structure of the magnetite, which is critical for maintaining the superparamagnetic and catalytic performance of the material in environmental applications.

**Figure 2:**
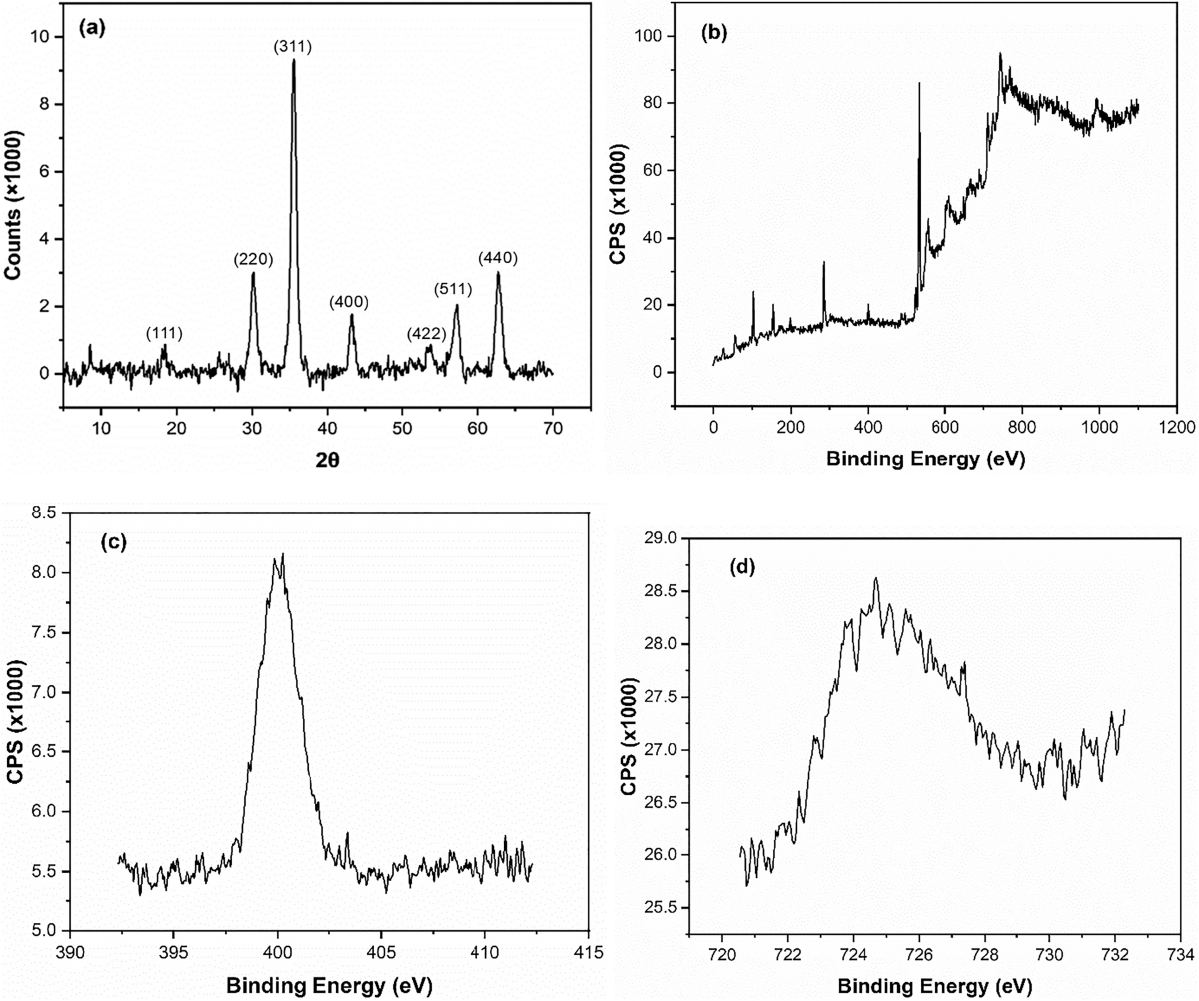
(a) XRD and (b) XPS results of Fe^2+^-Tpy@SPIONs catalyst; (c, d) represent the expansion of N 1s and Fe 2p_3/2_, respectively.

X-ray photoelectron spectroscopy (XPS) was carried out to elucidate the surface elemental composition and the oxidation states of key components in the Fe^2+^-Tpy@SPIONs catalyst. The wide-scan XPS spectrum (Figure 2b) confirmed the presence of Fe, O, C, N, and Si elements, verifying successful surface functionalization with the silica shell and organic ligands. To investigate the chemical environment of iron, high-resolution Fe 2p spectra (Figure 2d) were analyzed. Two prominent peaks appeared at binding energies of approximately 710.8 and 724.2 eV, corresponding to the Fe 2p_3/2_ and Fe 2p_1/2_ levels, respectively. These peaks, along with visible satellite features, indicate the coexistence of Fe^2+^ and Fe^3+^ oxidation states—consistent with the presence of magnetite (Fe_3_O_4_) as the magnetic core. This mixed-valence state is essential for maintaining the superparamagnetic behavior and catalytic redox activity of the material.

In addition to confirming the magnetite structure, XPS analysis also provided strong evidence of successful surface coordination between Fe^2+^ ions and the terpyridine ligands. The high-resolution N 1s spectrum (Figure 2c) exhibited a peak at ∼399.8 eV, which is characteristic of nitrogen atoms involved in metal–ligand coordination. This binding energy shift, relative to free terpyridine, confirms the formation of Fe^2+^–N bonds—indicating that the terpyridine ligand has successfully chelated Fe^2+^ on the nanoparticle surface. Such coordination not only stabilizes the iron species against oxidation and leaching but also plays a critical role in enabling the Fenton-like catalytic activity required for efficient PAH degradation. Thus, the XPS results comprehensively validate the chemical architecture of the Fe^2+^-Tpy@SPIONs catalyst and highlight the importance of ligand coordination in enhancing catalytic performance.

Fourier transform infrared (FTIR) spectroscopy and thermogravimetric analysis (TGA) were employed to evaluate the surface functionalization and thermal stability of the Fe^2+^-Tpy@SPIONs catalyst. The FTIR spectrum and TGA curve are shown in Figure 3a,3b, respectively. The FTIR spectrum exhibits several distinct absorption bands confirming the successful stepwise modification of the nanoparticle surface. A prominent peak at ∼580 cm^–1^ corresponds to Fe–O stretching vibrations, characteristic of the iron oxide core. The strong absorption band around 1100 cm^–1^ is attributed to Si–O–Si asymmetric stretching, confirming the presence of the silica shell. The absorption band at approximately 1620 cm^–1^ is assigned to the C=N stretching vibration of the terpyridine ligand, indicating its successful grafting onto the surface. Additional bands near 2900 cm^–1^ are due to C–H stretching vibrations from alkyl chains of the isocyanate linker. Furthermore, a broad band in the 3300–3400 cm^–1^ range suggests N–H stretching, consistent with the formation of an NH–CO–NH linkage between the isocyanate group and the amine of the terpyridine ligand. Notably, the absence of a peak near 2270 cm^–1^ (associated with −N=C=O stretching) indicates complete consumption of isocyanate groups, confirming the successful completion of the functionalization reaction.

**Figure 3:**
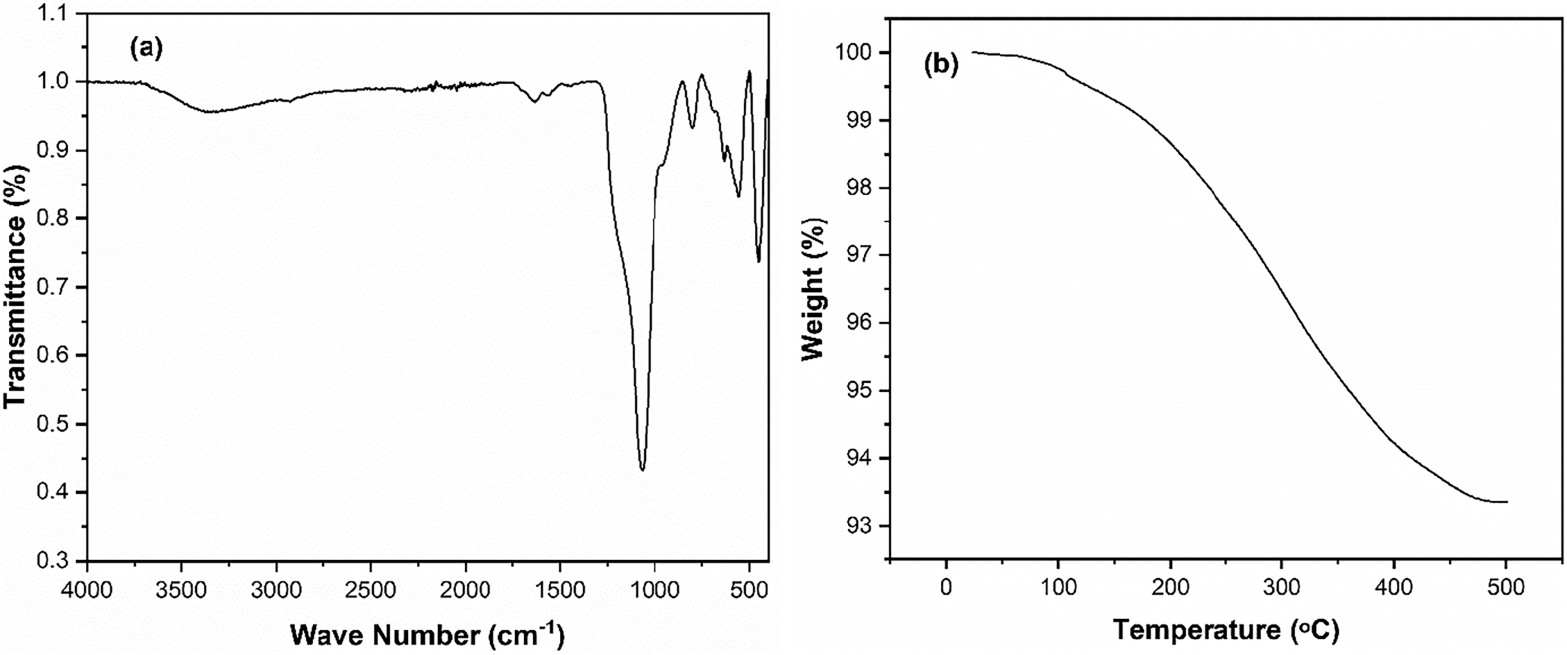
(a) FTIR and (b) TGA result of Fe^2+^-Tpy@SPIONs catalyst.

TGA was used to assess the thermal stability and the amount of organic content (terpyridine ligand) on the catalyst. The thermogram showed that the weight loss below 200 °C was negligible, indicating minimal loss of surface-bound water or residual solvents. A noticeable weight loss of approximately 8% was observed between 200 and 400 °C, which can be attributed to the decomposition of the organic terpyridine ligands grafted onto the nanoparticles. This gradual weight loss confirms the successful surface functionalization while demonstrating the high thermal stability of the Fe^2+^-Tpy@SPIONs catalyst up to 400 °C, making it suitable for high-temperature catalytic applications. The combined FTIR and TGA results strongly support the effective modification of the nanoparticles with terpyridine, a key factor in the enhanced catalytic performance of the system.

The optimization of the catalytic degradation of PAHs using Fe^2+^-Tpy@SPIONs was performed by varying key parameters, including pH, catalyst dosage, and temperature, to identify the most efficient reaction conditions. Through a series of controlled experiments, it was determined that the optimal degradation of PAHs occurred at a pH of 6.5, where the catalyst’s performance was maximized. At this slightly acidic pH, the Fe^2+^ ions remained stable and active in facilitating Fenton-like reactions, leading to the efficient generation of hydroxyl radicals for the oxidative breakdown of PAHs. Additionally, a catalyst concentration of 0.5 g/L was found to provide the best balance between catalytic activity and economic feasibility, ensuring sufficient surface area for pollutant interaction without excessive catalyst use.

Temperature optimization showed that the most effective degradation occurred at 25 °C, demonstrating that room temperature conditions are sufficient for high catalytic efficiency. Higher temperatures did not significantly enhance the degradation rate, suggesting that the Fe^2+^-Tpy@SPIONs catalyst is highly active even under mild conditions, making the process energy-efficient and practical for large-scale applications.

Together, these optimized parameters—pH 6.5, 0.5 g/L of catalyst, and 25 °C—represent the ideal conditions for achieving maximum PAH degradation while maintaining cost-effectiveness and operational simplicity. This highlights the robust performance of the Fe^2+^-Tpy@SPIONs system in real-world environmental remediation applications. The optimization results are presented in Table 2, and the removal of PAHs is calculated using eq 1

1 $$\mathrm{PAH}\;\mathrm{removal}=1-\frac{C_{i}-C_{f}}{C_{i}}\times 100$$

where *C*_f_ is the concentration of fluorene after removal by Fe^2+^-Tpy@SPIONs and *C_i_* is its initial concentration.

**Table 2:** Optimization Results of the Ability of Fe^2+^-Tpy@SPIONs in the Removal of Fluorene as a Model Compound for PAH Degradation

entry	pH	*T* (°C)	catalyst (mg)	removal (%)
1	8	25	500	88
2	7.5	25	500	91
3	7	25	500	90
4	6.5	25	500	98
5	6	25	500	96
6	6.5	50	500	98
7	6.5	75	500	98
8	6.5	25	400	88
9	6.5	25	600	98
10	6.5	25	800	98
11	6.5	25	1000	98
12	6.5	25	Tpy@SPIONs	9
13	6.5	25	SiO_2_@SPIONs	trace
14	6.5	25	No catalyst	trace

It should be noted that control experiments were conducted to confirm the necessity of the Fe^2+^-Tpy@SPIONs catalyst. When the reaction was performed using Tpy@SPIONs (without Fe^2+^) or SiO_2_@SPIONs as catalysts, no PAH removal was observed, highlighting the critical role of Fe^2+^ in driving the catalytic process. Additionally, in the absence of any catalyst, the PAH concentration remained unchanged, confirming that the degradation is directly attributed to the catalytic action of Fe^2+^-Tpy@SPIONs. These control experiments underscore the importance of Fe^2+^ immobilization and surface modification in achieving effective PAH degradation.

The application of the Fe^2+^-Tpy@SPIONs catalyst for the removal of PAHs from real produced water (PW) samples demonstrated its exceptional efficiency and practicality in a real-world context. In these tests, the total organic carbon (TOC) of the untreated PW sample was measured at 637 ppm, indicative of significant organic contamination, including persistent PAHs. After treatment with the Fe^2+^-Tpy@SPIONs catalyst under optimized conditions, the TOC was dramatically reduced to 115 ppm, representing an 82% reduction in the total organic load. This substantial decrease in TOC underscores the catalyst’s high effectiveness in degrading complex organic pollutants, even in challenging wastewater samples like produced water.

The remarkable performance of the Fe^2+^-Tpy@SPIONs catalyst can be attributed to its unique combination of magnetic recoverability, efficient Fe^2+^ coordination with the terpyridine ligand, and the generation of hydroxyl radicals through Fenton-like reactions. This high catalytic efficiency was achieved under mild conditions, at room temperature (25 °C), with minimal catalyst loading (0.5 g/L), making the process both cost-effective and environmentally friendly. The ability to remove a significant proportion of organic contaminants from real produced water (PW) samples demonstrates the practical applicability of Fe^2+^-Tpy@SPIONs for large-scale environmental remediation in the oil and gas industry. This catalyst not only provides a sustainable solution for treating industrial wastewater but also significantly reduces the environmental impact of produced water discharge, marking a breakthrough in advanced catalytic systems for water purification. It should be noted that produced water is a complex matrix that contains not only polycyclic aromatic hydrocarbons (PAHs) but also high concentrations of salts, heavy metals, and other organic and inorganic species. Although the primary focus of this study was PAH degradation, the significant reduction in total organic carbon (TOC) from 637 to 115 ppm in real PW samples suggests that the Fe^2+^-Tpy@SPIONs catalyst can oxidize a broad spectrum of organic contaminants. However, PAHs were chosen as model pollutants due to their environmental relevance and persistence. While we did not isolate and quantify individual organic species in real PW, the high degradation efficiency in both model and real systems indicates the catalyst remains active under complex conditions. Future work will focus on evaluating selectivity by identifying degradation intermediates and assessing the role of competing species such as chloride ions or heavy metals in the catalytic process.

The lower removal efficiency observed in real produced water (∼82% TOC reduction compared to ∼98% fluorene degradation) may be attributed to several factors. First, the higher organic load (∼600 ppm) may approach the catalyst’s capacity, leading to partial saturation or reduced activity. Second, produced water contains a complex mixture of organic compounds beyond PAHs, some of which may be more recalcitrant to oxidation or may inhibit catalytic sites. While we did not perform a second-stage treatment or detailed analysis of the residual organics in this study, future work will investigate whether adding a fresh dose of catalyst or increasing the oxidant concentration can further reduce TOC. Additionally, identifying the remaining organic species could help determine if incomplete mineralization is due to compound-specific resistance or catalyst limitations.

The reusability of the Fe^2+^-Tpy@SPIONs catalyst was evaluated over five consecutive cycles of PAH degradation to assess its long-term stability and catalytic performance. After each catalytic cycle, the catalyst was magnetically separated from the reaction mixture, washed thoroughly with deionized water and ethanol, and then dried under vacuum for reuse in subsequent cycles.

The catalytic efficiency of Fe^2+^-Tpy@SPIONs remained consistent across the five cycles, with minimal loss in PAH removal efficiency, highlighting the robustness of the catalyst. The recovery results from the five cycles are presented in Figure 4, demonstrating the stability and reusability of the catalyst as evidenced by the recovery data.

**Figure 4:**
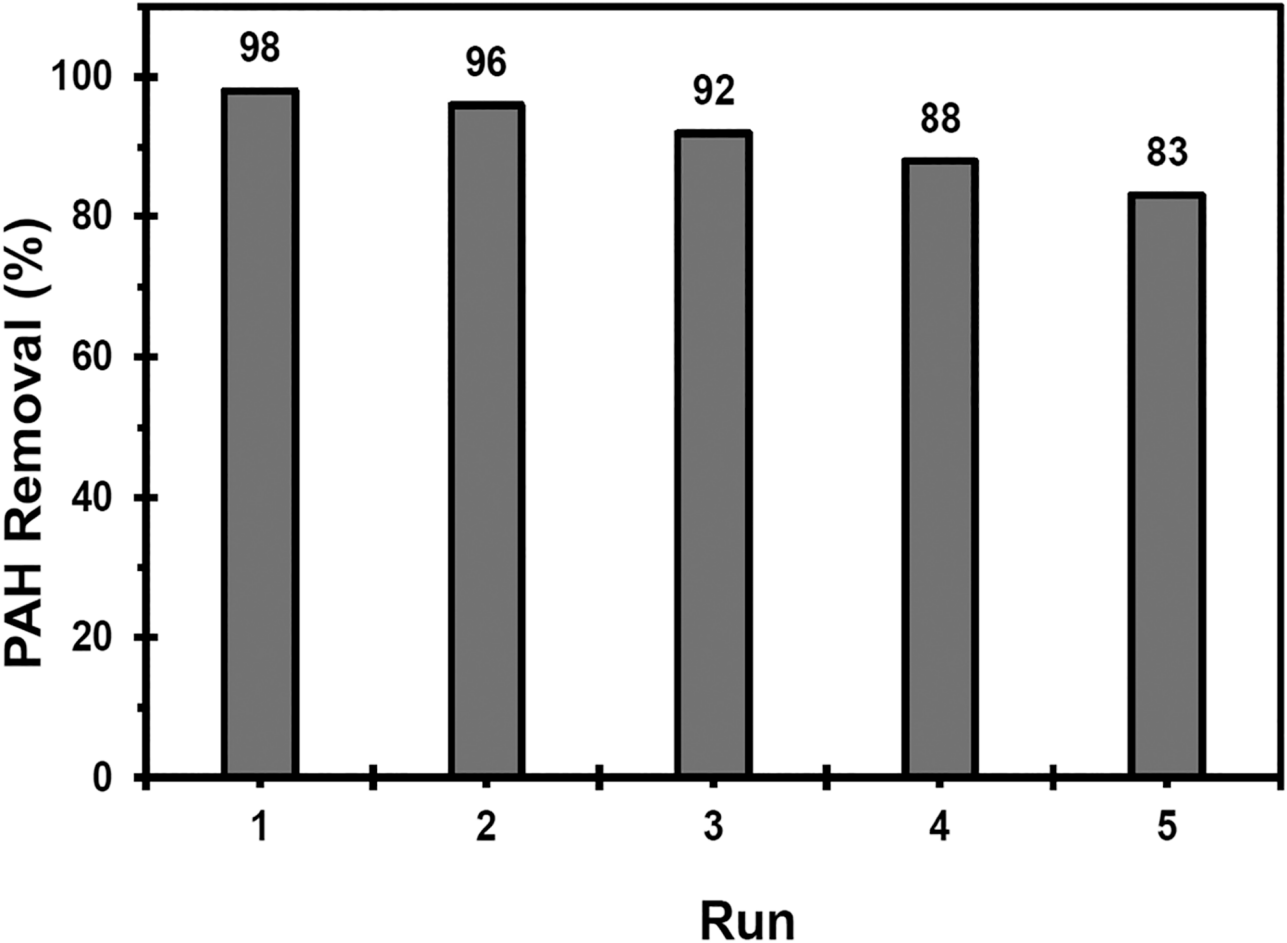
Recovery results of Fe^2+^-Tpy@SPIONs catalyst in the removal of simulated PAH.

To further investigate the structural and chemical stability of the recovered catalyst after five cycles, it was characterized using SEM and FTIR spectroscopy, as shown in Figure 5a,b, respectively. SEM images confirmed that the nanoparticles retained their spherical morphology and uniform size distribution, with no significant aggregation or degradation observed. The average particle size remained approximately 25 nm, indicating that the structural integrity of the Fe^2+^-Tpy@SPIONs catalyst was maintained throughout repeated use.

**Figure 5:**
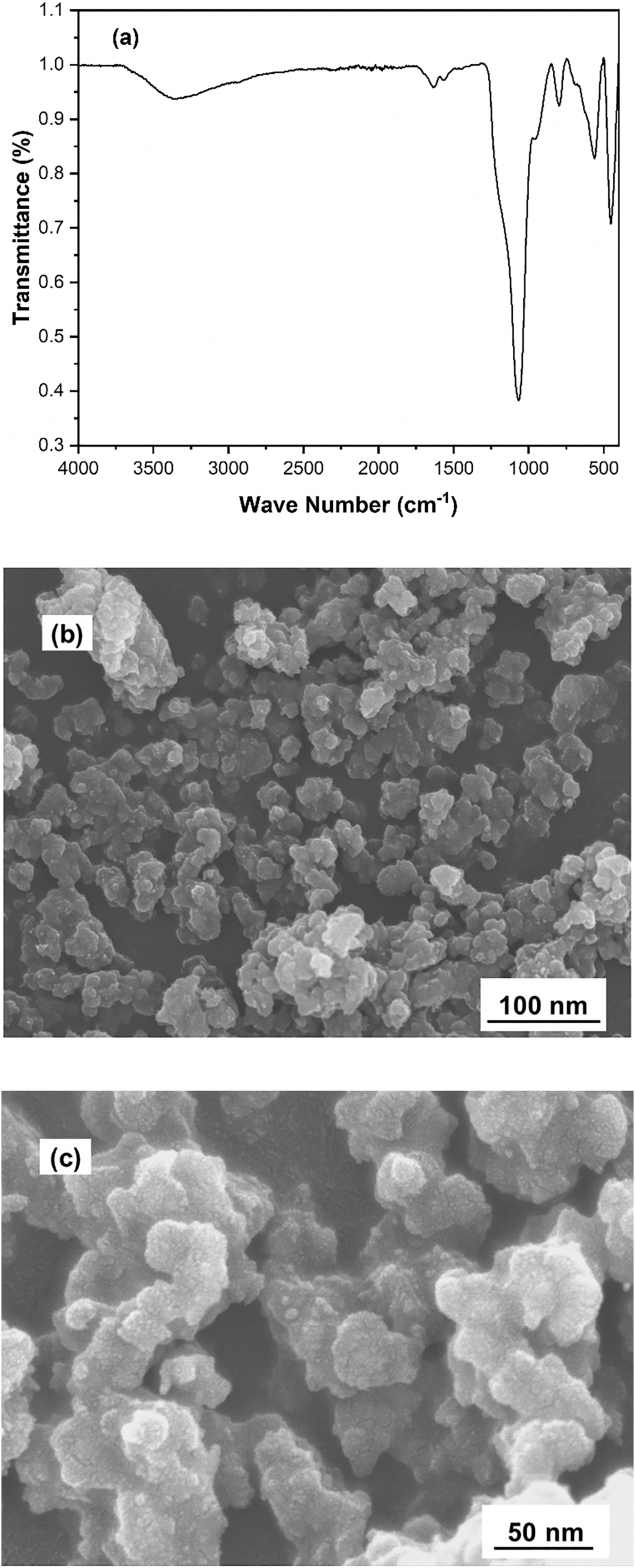
(a) FTIR spectrum and (b, c) SEM result of Fe^2+^-Tpy@SPIONs catalyst with different magnifications after recovery from five cycles.

FTIR analysis of the recycled catalyst revealed that the characteristic absorption bands associated with the terpyridine ligand (around 1620 cm^–1^ for C=N stretching) and the silica shell (around 1100 cm^–1^ for Si–O–Si stretching) were still present, confirming that the surface functionalization and Fe^2+^-Tpy coordination remained intact after multiple cycles. The retention of these functional groups indicates that the catalyst’s active sites were preserved, which is crucial for maintaining its catalytic performance. These findings demonstrate that the Fe^2+^-Tpy@SPIONs catalyst is not only highly efficient in degrading PAHs but also exhibits excellent reusability and stability, making it a viable solution for large-scale, sustainable water treatment applications.

Based on the structural features of Fe^2+^-Tpy@SPIONs and previous literature on Fenton-like systems, a plausible mechanism for PAH degradation is proposed (Scheme 2). The catalytic cycle is initiated when Fe^2+^ ions, immobilized on the nanoparticle surface via terpyridine ligands, react with hydrogen peroxide (H_2_O_2_) to generate highly reactive hydroxyl radicals (^•^OH) according to the classical Fenton reaction. These hydroxyl radicals attack PAH molecules in solution, leading to hydroxylation, ring opening, and eventual mineralization into CO_2_ and H_2_O. The generated Fe^3+^ is then reduced back to Fe^2+^, either by excess H_2_O_2_ or by organic intermediates present in the solution, thus completing the redox cycle. The terpyridine ligand plays a dual role: it stabilizes Fe^2+^ on the surface and facilitates its redox cycling by maintaining a favorable coordination environment. This surface-bound Fe^2+^ ensures localized radical generation near the nanoparticle, enhancing the degradation efficiency. The effectiveness of the catalyst under mild conditions suggests that the Fe^2+^–Tpy coordination is critical for sustaining catalytic activity and suppressing Fe leaching, which often limits conventional Fenton systems.

**Scheme 2:**
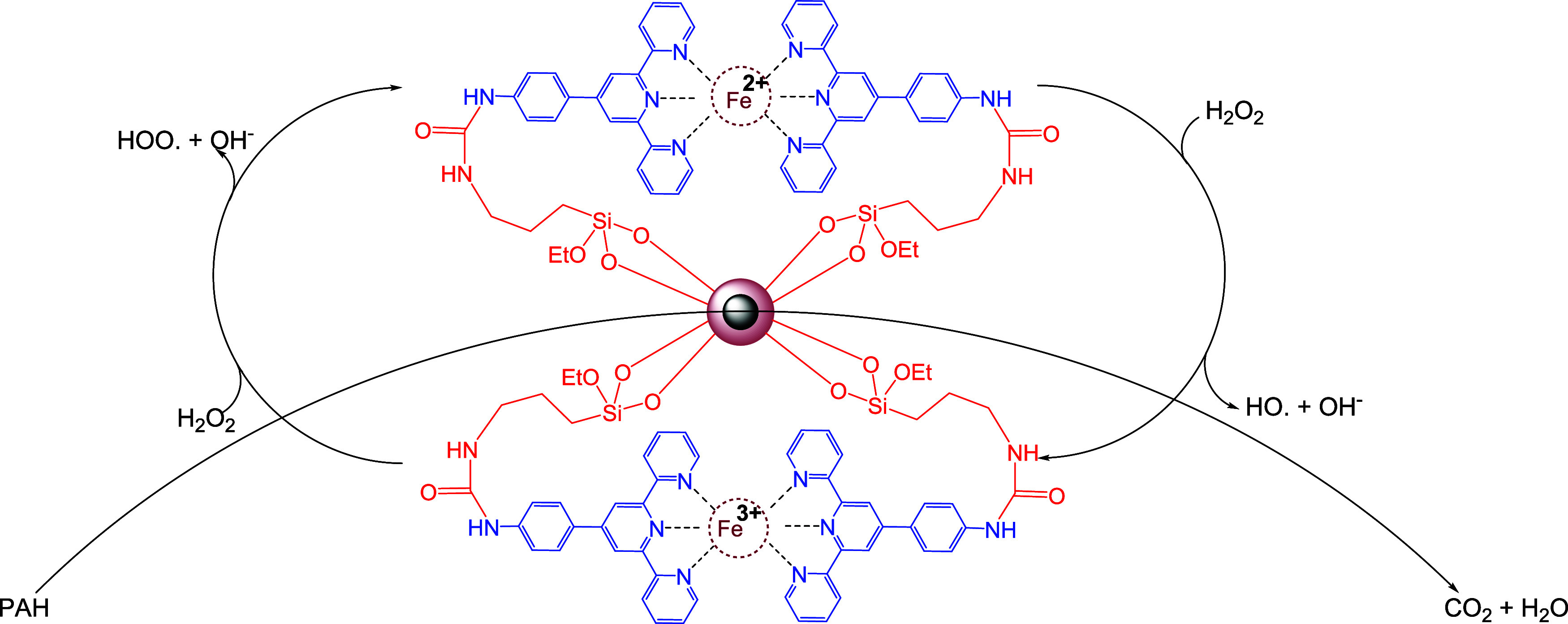
Proposed Mechanism for the Oxidative Reaction of PAH Removal in the Presence of Fe^2+^-Tpy@SPIONs

Table 3 compares the PAH removal efficiencies of Fe^2+^-Tpy@SPIONs catalyst with previously published systems. As shown, while many systems achieve PAH removals, most of them either depend on high catalyst dosages, nonrecyclable adsorbents, or operate under conditions that may not translate to produced water matrices. By contrast, our system combines a high TOC removal (82%) with recyclability, mild operational conditions, and magnetic recoverability.

**Table 3:** Comparison of the Efficiency of Fe^2+^-Tpy@SPIONs Catalyst with Previously Reported Systems

catalyst	conditions	recyclability	PAH removal (%)	refs
ferrate (VI)	workup at pH 12.0	no	89.73	^53^
microbial	use of microorganisms	no	53.4–100	^54^
nanozero-valent iron	photosensitive reaction	no	75.3–89.5	^55^
ZnO-TiO_2_	photosensitive reaction	no	25–78	^56^
Fe^2+^-Tpy@SPIONs	current work	yes	82%	

## 4 Conclusion

In this study, the Fe^2+^-Tpy@SPIONs catalyst was developed and successfully applied for the efficient removal of PAHs from produced water, demonstrating its potential as a robust and sustainable solution for environmental remediation. The unique combination of superparamagnetic iron oxide nanoparticles, terpyridine surface functionalization, and Fe^2+^ coordination resulted in a highly active and magnetically recoverable catalyst capable of effectively degrading PAHs under mild reaction conditions.

Optimization of key parameters, including pH, catalyst dosage, and temperature, revealed that the best catalytic performance was achieved at pH 6.5, a catalyst concentration of 0.5 g/L, and 25 °C, making the process energy-efficient and scalable for industrial applications. Real-world applicability was confirmed through experiments on actual produced water samples, where the catalyst reduced the total organic carbon (TOC) from 637 to 115 ppm, achieving an impressive 82% reduction in organic contaminants.The catalyst’s reusability was further validated, with consistent performance over five cycles while maintaining its structural integrity and surface functionality, as confirmed by SEM and FTIR analyses.

Overall, the Fe^2+^-Tpy@SPIONs catalyst represents a significant advancement in water treatment technologies, offering a cost-effective, recyclable, and highly efficient approach for the degradation of hazardous organic pollutants in produced water. This study paves the way for the broader adoption of advanced nanoparticle-based catalysts in environmental remediation, addressing critical challenges in wastewater management across the oil and gas industry and beyond. While hydrogen peroxide is a widely used and effective oxidant in Fenton-like reactions, its large-scale application for treating billions of barrels of produced water poses economic and logistical challenges. The costs associated with the supply, storage, and handling of H_2_O_2_ must be carefully evaluated for industrial deployment. However, several strategies—such as reducing H_2_O_2_ dosage through kinetic optimization, generating H_2_O_2_ in situ, or employing alternative systems like solar- or electro-Fenton processes, offer promising pathways to enhance cost-efficiency. It is important to emphasize that the present study serves as a proof of concept to demonstrate the feasibility and catalytic performance of the Fe^2+^-Tpy@SPIONs system. To support scalability, we have intentionally employed low-cost precursor materials such as iron chloride, which are abundant and industrially accessible, making this approach potentially viable for high-volume produced water treatment. Overall, the superior recyclability, high catalytic efficiency under near-neutral conditions, and enhanced stability provided by the Fe^2+^–terpyridine coordination clearly set Fe^2+^-Tpy@SPIONs apart from conventional Fenton-like catalysts, underscoring their novelty and practical relevance for industrial wastewater treatment.
